# Theoretical and Practical Implications of Treating Cachexia in Advanced Lung Cancer Patients

**DOI:** 10.3390/cancers11111619

**Published:** 2019-10-23

**Authors:** Philip Bonomi, Mary Jo Fidler, Palmi Shah, Jeffrey Borgia

**Affiliations:** 1 Division of Hematology/Oncology, Rush University Medical Center, Chicago, IL 60612, USA; Mary_fidler@rush.edu; 2 Department of Radiology, Rush University Medical Center, Chicago, IL 60612, USA; Palmi_shah@rush.edu; 3 Department of Cell & Molecular Medicine, Rush University Medical Center, Chicago, IL 60612, USA; Jeffrey_A_Borgia@rush.edu; 4 Department of Pathology, Rush University Medical Center, Chicago, IL 60612, USA

**Keywords:** lung cancer, cachexia, immunotherapy, trial endpoints

## Abstract

Lung cancer continues to be a major worldwide health issue, with more than 50% of patients having incurable metastatic disease at diagnosis. Fortunately, the advanced lung cancer treatment landscape is changing rapidly as a result of the positive impact of effective inhibitors of tumor driver mutations, and the more recent discovery that immune modulation with anti-PD-1/PD-L1 monoclonal antibodies results in tumor regression and prolonged survival. While a relatively small subset of lung cancer patients are candidates for inhibitors of driver mutations, the majority of advanced lung cancer patients are candidates for an immunotherapy regimen. Many of these patients have cachexia, which is associated with increased cancer therapy toxicity and possibly reduced responsiveness to immunotherapy. Two ongoing cachexia trials, one testing a ghrelin analogue and the other testing a multimodal strategy, have endpoints which assess clinical benefit—weight gain and relief of anorexia/cachexia symptoms. Provided that the trial objectives are achieved, these treatment strategies will provide a way to relieve suffering and distress for cachectic cancer patients. While awaiting the results of these trials, it would be reasonable to consider designing studies testing cachexia treatments combined with first-line immunotherapy and chemotherapy–immunotherapy in stage IV lung cancer patients, with enhanced overall survival being one of the endpoints.

## 1. Introduction

Cachexia is common in lung cancer, occurring in almost 50% of patients with locally advanced or widespread disease, and it occurs more frequently in cancer patients with higher stages of disease [1]. It is likely that increased implementation of screening chest CT scans [2] will reduce the percentage of lung cancer patients presenting with late stage tumors. Currently, approximately 80% of patients have locally advanced or metastatic disease at diagnosis [3]. Since it will take a significant amount of time for lung cancer screening to result in a decrease in late-stage lung cancer at diagnosis, it is likely that cancer cachexia will continue to be a significant clinical problem for the majority of lung cancer patients.

Prior to 2004, cytotoxic chemotherapy alone or combined with chest radiation for locally advanced disease was the primary treatment option for this large group of patients [4]. However, with the discovery of driver-mutation-specific targeted therapies (*EGFR*, *ALK*, *ROS1*) and recent FDA approvals for the use of immune checkpoint inhibitors, major paradigm shifts have occurred [5,6]. It is now the standard of care to begin a tyrosine kinase inhibitor for patients with *EGFR*, *ALK*, *ROS-1* and other actionable mutations if known via molecular profiling prior to the initiation of chemotherapy. In patients without an actionable driver mutation, immune checkpoint inhibitors targeting the *PD-1*/*PD-L1* immune checkpoints [5,6] have become the standard first line treatment for advanced non-small cell lung cancer (NSCLC) patients [7,8,9,10]. Recent data also show a survival benefit of adding atezolizumab to platinum/etoposide for extensive-stage small cell lung cancer [11], and durvalumab after chemotherapy and radiation for locally advanced NSCLC [12].

Most advanced lung cancer patients are potential candidates for initial treatment with one of the new immunotherapy regimens [7,8,9,10] However, many lung cancer patients do not receive systemic treatment for a variety of reasons, with poor performance status being among the most common [13] Although there is relatively little information regarding the relationship between performance status and cachexia, there has been at least one report which described significantly worse Karnofsky performance status, endurance capacity, and maximum power output in cachectic cancer patients [14]. In addition, there is also evidence that cachexia as defined at the International Cachexia Conference in 2011 [15] is associated with increased chemotherapy toxicity [16,17], and there is preliminary evidence that cachexia is associated with worse outcomes in patients treated with anti-*PD1*/anti-*PDL1* monoclonal antibodies [18,19]. It is likely that cachexia is a contributing factor to lung cancer patients not initiating or not tolerating systemic treatment, and it is conceivable that an effective, rapidly acting cachexia treatment combined with the new immunotherapy or chemotherapy/immunotherapy regimens might improve outcomes in advanced lung cancer patients. Here, we discuss potential theoretical implications of studying cachexia treatment strategies [20,21,22,23,24] in advanced lung cancer patients who are receiving one of the recently approved anti-*PD1*/anti-*PDL1* monoclonal-antibody-containing regimens [7,8,9,10,11,12], as well as practical considerations regarding clinical trial design and patient selection.

## 2. Cachexia Treatment and Implications for Survival: Pre-Clinical and Clinical Studies

The primary goal of treating cancer related cachexia has been to palliate symptoms associated with this metabolic disorder. With increasing understanding of the mechanisms of cachexia [25], it is likely that effective anti-cachexia treatments will be developed and receive regulatory approval. Based on pre-clinical studies, it is conceivable that the reversal of cachexia could have an independent effect on overall survival. There have been at least three studies in murine tumor cachexia models which have shown that successful treatment of cachexia was associated with superior survival [24,26,27]. Two of these groups of investigators studied colon cancer 26 cell lines injected into mice [26,27]. In one of the studies, an activin receptor llB inhibitor prevented skeletal muscle loss, and, while not preventing tumor progression, treatment with the ActRllB inhibitor was associated with significantly longer survival [25]. Administration of a histone deacetylase inhibitor, AR-42, to mice bearing colon tumor AR42 also reversed cachexia and was associated with superior survival [27].

More recently, an anti-growth differentiating factor 15 (GDF 15) monoclonal antibody was tested in a breast cancer murine model [24]. These investigators included an anti-neoplastic treatment consisting of tivozanib, a vascular endothelial growth factor receptor (VEGFR) tyrosine kinase inhibitor, in their experiments. The following treatments were compared: IgG alone versus IgG combined with tivozanib versus anti-GDF 15 monoclonal antibody combined with tivozanib. Treatment with the anti-GDF 15 monoclonal antibody successfully reversed cachexia and was also associated with superior survival compared to IgG alone and IgG plus tivozanib [24].

Conversely, observations in pre-clinical models may not be consistent with the mechanisms of cachexia in patients, and it has been argued that progress in treating cancer cachexia will require well designed clinical trials and concomitant laboratory correlates to identify causal mechanisms and potential therapeutic targets [25]. A recent review of interventional cachexia studies identified 65 randomized trials [28]. While some studies were limited to a single cancer type, the majority of these trial accrued patients with multiple types of cancer. Interventions included pharmacological agents, nutritional supplements, exercise, psychosocial support, or various combinations of these interventions. Study endpoints included body mass, nutritional status, physical function, symptoms, quality of life, and overall survival. Positive overall survival results were seldom observed in the 32% of randomized studies that reported survival results [28].

Survival results were described in two recently reported randomized cachexia trials [29,30]. One of the studies included 263 incurable cancer patients with multiple types of cancer and recent weight loss [29]. They were randomized to receive carnitine versus placebo. No significant differences were observed for overall survival or for cachexia parameters. The other trial enrolled 125 evaluable stage 2–4 pancreatic cancer patients with weight loss during the previous six months [30]. Patients were treated with chemotherapy and were randomly assigned to treatment with either two dose levels of the antimyostatin antibody or with placebo. Although there was a trend towards increased muscle mass and functional performance in the patient sub-group with baseline weight loss < 5%, the differences in muscle mass and physical performance for this sub-group and the entire group were not significant. Similarly, overall survival rates between antimyostatin- and placebo-treated patients were not significantly different [30].

Unlike in the pre-clinical studies [24,26,27], in both clinical studies [29,30], the intervention was not associated with a significant positive impact on cachexia [29,30]. For cachexia treatment to have a positive impact on overall survival in cancer patients, we believe that significant reversal of cachexia is essential. While positive correlations between the reversal of cancer cachexia and prolongation of survival have been observed in preclinical models, it is noteworthy that there have been a limited number of positive pre-clinical reports and only two cell lines have been studied [24,26,27]. Evaluation of the potential relationship between cachexia treatment and overall survival in advanced cancer patients will require a carefully designed clinical trial and enhanced understanding of cancer cachexia mechanisms [25]. However, while ongoing pre-clinical and translational studies are evaluating cachexia mechanisms, it would not be unreasonable to conduct a phase 2 trial with an intervention proven to reverse objective measures of cachexia and to look for a survival signal.

## 3. Cachexia Treatment and Implications for Cancer Treatment Toxicity

A relationship between sarcopenia and increased toxicity from cytotoxic agents was reported as early as 2009 [31]. Significantly higher rates of mucositis and diarrhea were observed in sarcopenic breast cancer patients treated with capecitabine. Although conducted in a relatively small number of patients, this observation suggests that cachexia enhances the risk of toxicity in tissues with rapid cell turnover. More recently, higher drug doses per kilogram of lean body mass were shown to be significantly related to increased hematological toxicity from carboplatin doublet regimens in 424 advanced non-small cell lung cancer patients treated in clinical trials [16]. In their multivariate analysis, a higher dose of the non-platinum drug in the chemotherapy doublets was significantly related to increased grade 3/4 hematological toxicity. Cytotoxic agents are currently dosed based on body surface area. The authors suggested that chemotherapy dosing based on body composition be evaluated in future clinical trials [28].

A retrospective study evaluated potential relationships between platinum chemotherapy delivery in head and neck cancer patients receiving radiation and concurrent platinum treatment [17]. Skeletal muscle index was measured at the fourth thoracic and third lumbar vertebrae. Both univariate and multivariate analyses revealed that lower skeletal muscle indices were significantly associated with higher odds of early termination of chemotherapy. The investigators suggested that treatment be tailored for patients with low muscle mass in order to avoid early termination of chemotherapy [17].

The number of studies describing relationships between cachexia and toxicity from cancer treatments is relatively small [16,17,31]. It is possible that increased toxicity from cancer treatments is related to pharmacokinetic and pharmacodynamic differences between cachectic versus non-cachectic patients. However, with the exception of one trial which showed more rapid clearance of pembrolizumab, which appeared to be related to cachexia [19], we are not aware of reports describing cancer treatment pharmacokinetics or pharmacodynamics in cachectic versus non-cachectic cancer patients. It conceivable that body composition changes related to cachexia could affect drug distribution. It is also possible that normal tissues with rapid cell turnover might be more sensitive to the cytotoxic effects of cancer treatments as a result of ongoing catabolism associated with cachexia [1].

Previous groups of investigators [16,17] have recommended considering dose modulation for cancer patients with low muscle mass. Although not based on body composition measurements, our group has routinely reduced chemotherapy doses by at least 20% in patients with metastatic lung cancer and recent weight loss and/or low serum albumin. In locally advanced lung cancer patients, we have not only reduced the dose of chemotherapy, we have also utilized split course chest radiation [32], as opposed to the conventional use of uninterrupted chest radiation. While we agree with dose modulation, it is also conceivable that incorporating an agent which effectively reverses cachexia might also reduce toxicity and increase patients’ opportunity to receive anticancer therapy.

In order to be useful, reversal of cachexia should be rapid. Anamorelin is a ghrelin receptor agonist which increased lean muscle mass in two randomized phase III trials [19]. An agent like anamorelin, which was associated with increase in weight and increase appetite within three weeks [19], might impact toxicity and treatment delivery for stage IV lung cancer patients receiving cytotoxic agents, as well as for stage III lung cancer patients receiving cytotoxic agents and concurrent chest radiation. If concurrent cachexia treatment enables the completion of cancer treatments, it is conceivable that overall survival might be impacted by this combined modality approach.

## 4. Cachexia Treatment and Potential Implications for Outcomes on Immunotherapy

Currently, the majority of advanced lung cancer patients are candidates for first-line systemic therapy regimens which include immune checkpoint inhibitors. There is preliminary evidence that the mechanisms involved in cachexia are associated with worse outcomes in lung cancer patients treated with immune checkpoint inhibitors. French investigators evaluated survival outcome in 251 consecutive cancer patients treated in phase 1 trials testing anti-*PD1*/PDL1 monoclonal antibodies [18]. The most common diagnoses in their studies were melanoma and lung cancer. Low skeletal muscle index measured at the third lumbar vertebra (53 cm^2^/m^2^), along with high tumor burden and extrapulmonary metastatic disease, were associated with associated significantly shorter survival [18].

Another group conducted analyses to evaluate the potential relationship between overall survival and pembrolizumab dose and exposure. This humanized IgG4 monoclonal antibody targeting PD-1 was administered to 804 non-small cell lung cancer patients and 340 melanoma patients who had participated in clinical trials comparing two doses of pembrolizumab [19]. While they found no significant relationships between overall survival and dose of pembrolizumab or exposure to pembrolizumab, they found that decreasing body weight and decreasing serum albumin level during treatment were significantly associated with shorter overall survival during treatment. They also observed that more rapid baseline clearance of pembrolizumab was associated with significantly shorter survival. They postulated that their observations were due to patients with ongoing cachexia having a higher rate of catabolism, which resulted in more rapid clearance of the anti-PD1 monoclonal antibody. They also advocated for identification of better predictive biomarkers of cachexia as a way to identify cancer patients who might be less likely to benefit from immunotherapy [19].

Reports from these investigators [18,19] suggest that additional study of the relationship between cachexia and response to anti-*PD1*/*PDL1* monoclonal antibodies is warranted. Pre-clinical and translational studies evaluating the interaction of cytokines and cells involved in cachexia and the innate immune response are needed to elucidate potential therapeutic interventions [18,24]. Meanwhile, the preliminary clinical [18] and pharmokinetic [19] observations suggest that it is reasonable to conduct a single-arm or small, randomized phase II clinical trial testing an agent that rapidly reverses cachexia combined with an immune checkpoint inhibitor in advanced lung cancer patients. Since a ghrelin analogue has been shown to rapidly reverse weight loss in lung cancer patients [20] and ghrelin increased T-cell proliferation in a pre-clinical study [33], the currently available ghrelin analogue could be studied in this type of trial. Collection of patients’ blood and tissue specimens for molecular and cellular correlative studies would be an essential component of such a study.

## 5. Endpoints for Cachexia Trials

The complexity of defining endpoints for cachexia trials is highlighted by the lack of agreement regarding these measures, which have primarily included body composition, functional status, and nutrition [34]. Rather than providing a comprehensive review of the endpoints included in previous trials, we focused on endpoints which are accepted as indicators of clinical benefit, and therefore are relevant for regulatory approval. Randomized double blind trials of anamorelin (a ghrelin agonist) and enobosarm (a selective androgen receptor modulator) in NSCLC patients were recently reported [20,21]. The anamorelin studies [20] evaluated all three end points, whereas the enobosarm trials measured body composition and physical performance [21]. There were significant differences in the type and timing of cancer treatment regimens in each trial. In the anamorelin studies, cancer treatment regimens included a variety of chemotherapy treatments, radiation therapy, or supportive care only [20]. In contrast, in the enobosarm studies, treatment was limited to first-line platinum regimens, with one group of patients being treated with taxane/platinum and the other group being treated with a non-taxane/platinum [21]. Despite differences in cancer treatment, the studies showed that the cachexia treatment had a significant biological effect of increased lean muscle mass [20,21], suggesting that if the primary endpoint in a cachexia trial is change in body composition, it appears that permitting multiple types and lines of cancer treatment is appropriate and might enhance accrual.

Cancer treatment on its own, however, appears to impact weight change in advanced NSCLC patients during treatment with first line chemotherapy. In a retrospective study comprising 2300 advanced stage NSCLC patients treated with first-line platinum regimens in phase III clinical trials, 18.5% of the patients gained > 5% of their baseline weight [35]. This observation suggests that an imbalance in the number of patients receiving first-line therapy versus greater-than-second-line systemic therapy might confound comparison of the changes in weight/lean body mass in cachexia trials.

We agree with the statement that increased body weight is an important indicator of clinical benefit in cancer patients [34]. Maintaining or increasing body weight is extremely important to a patients’ sense of well-being, and it reduces a source of stress between them and their loved ones. Anamorelin is currently being studied in two randomized, placebo-controlled trials (NCT 03743064, NCT 03743051) in NSCLC patients, with the primary endpoints of weight gain and improvement in anorexia related symptoms. There is also an ongoing trial (MENAC, Eudra CT 2013-102282-19) testing a multimodal strategy including nutritional supplements, exercise, and a non-steroidal anti-inflammatory agent in lung cancer and select GI cancers, with the primary endpoint of weight gain [22]. Table 1 includes the above trials and additional ongoing interventional cachexia trials listed under the U.S. National Library of Medicine, Clinical Trials.gov, and under the EU Clinical Trials Register, eudract.ema.europa.eu. Recently completed interventional cachexia trials have been described in a recent review [1].

Despite increasing lean body mass, amamorelin and enobosarm did not meet the functional endpoints of hand grip strength and stair climb, respectively [20,21]. Trying to determine the best way to measure improvement in physical performance is difficult. Intuitively, we would expect increased muscle mass to be associated with increased performance. However, it is likely that increase in muscle mass in healthy individuals is related to ongoing physical training. It seems unlikely that increased performance will occur without physical training. Neither trial [20,21] included physical training as part of their protocol. The MENAC trial’s multimodal strategy, which requires exercise on a regular basis [22], should provide information regarding the impact of physical training. Measuring typical daily activities might be a more meaningful indicator of patient function [36]. This parameter might be measured with patient “wearables”, or biometric devices [37], and could measure something as simple as counting the number of steps that patients have taken between assessment periods. This methodology requires validation in cancer patients, starting with assessment of patient compliance.

Another cachexia endpoint is nutritional status, which is determined by food intake, absorption, assimilation, and anabolism/catabolism balance. While collecting information regarding nutrient intake and resting energy expenditure is interesting and important, we believe that a validated anorexia/cachexia quality of life instrument [38] would provide clinically meaningful information regarding patients’ symptoms which impact nutritional status. Anorexia is a major component of cancer patients’ suffering and a source of conflict between patients and the caregivers. We agree with the opinion that clinicians who provide care for anorectic cancer patients consider relief of anorexia–cachexia symptoms to be an important indicator of clinical benefit [34]. FDA physicians also agree, and have recommended obtaining patient reported outcomes related to disease related symptoms as an important endpoint for cancer treatment trials [39]. Assessing anorexia–cachexia patient reported outcomes is a primary endpoint in the ongoing anamorelin randomized trials (NCT 03743064, NCT 03743051).

## 6. Patient Selection in Lung Cancer Trials

Should future cachexia trials be limited to patients who have cachexia as defined by the criteria established at the consensus conference in 2011 [15], or should patients with pre-cachexia (<5% weight loss) also be included in future studies? While establishing the criteria for cachexia was an important achievement, previous weight loss information is usually subjective. With increasing use of electronic records and emphasis on the importance of weighing patients at every clinic visit, there should be access to real-time body weight data for most patients. A grading system for cancer-associated weight loss, which was based on both percent recent weight loss and body mass index [40], was developed and validated in large cohorts of patients. Individuals were assigned to one of five grade levels, and the findings of this study indicated that specific grade levels were associated with significantly different survival rates. Selecting patients with a weight loss/BMI score of 2 or 3 as defined by this grading system might enable the inclusion of patients who are in the early phases of cachexia and who might be more likely to benefit from cachexia treatment [15,36]

Finally, complete blood counts are done in every cancer patient and might provide a simple way to select patients for cachexia trials. A higher neutrophil lymphocyte ratio (NLR) has been associated with inferior survival in multiple types of cancer [41,42]. In a small retrospective study, increasing longitudinal NLRs were associated with decreasing weight [43], suggesting that high NLR may be a means by which to identify patients who are experiencing the early stages of cachexia without meeting the strict definition of cachexia [15]. If additional data are consistent with this initial observation, high NLR could be considered as an eligibility criterion for future cachexia studies. It should be noted that NLR may not have prognostic significance in patients receiving corticosteroids.

## 7. Summary

Lung cancer continues to be a major worldwide healthcare issue, and the majority of lung cancer patients will experience anorexia–cachexia during the course of their illness. We believe that continuing to study cachexia treatments in advanced non-small cell lung cancer patients is an important strategy. We await the results of the anamorelin and the MENAC trials. If reversal of weight loss and reduction of anorexia is observed in these studies, these interventions will relieve major sources of suffering for many lung cancer patients. In the rapidly changing lung cancer treatment landscape, immunotherapy alone [7] or in combination with chemotherapy [8,9,10] is associated with superior survival. Many of these patients have cachexia or pre-cachexia. While studies attempting to identify effective cachexia treatments and efforts to elucidate the molecular and cellular relationships between cachexia and immune response are ongoing, it is reasonable to consider developing trials to evaluate the potential impact of cachexia treatment combined with immunotherapy regimens on overall survival in cachectic and possibly pre-cachectic advanced lung cancer patients. With the high prevalence and aggressive nature of lung cancer, results from lung cancer cachexia trials should be available relatively quickly, and could have implications for cachectic patients with other types of malignancies.

## Figures and Tables

**Table 1:** Current Interventional Cachexia Trials.

Study Identifier	Randomized	Cancer Type	Accrual Target	Status	Intervention
NCT 03740351	Yes	Non-small cell lung cancer	316	Recruiting	Anamorelin Hydorchloride
NCT 03743064	Yes	Non-small cell lung cancer	316	Recruiting	Anamorelin Hydrochloride
Eudra CT 2012-002282-19	Yes	Lung/pancreatic cancers	240	Recruiting	Nutrition, exercise, ibuprofen
NCT 03283488	Yes	Multiple cancers	52	Not yet recruiting	Megestrol/Mirtazepine
NCT 01614990	Yes	Multiple cancers	8	Recruiting	Macimorelin
NCT 03207724	No	Pancreatic cancer	16	Recruiting	Xilonix/Onivyde/5FU
NCT 03631459	No	Lung/GI cancers	100,000	Not yet recruiting	Kanglaite
NCT 04065815	No	Multiple cancers	100	Recruiting	Exercise, protein Supplement
NCT 03720158	Yes	Head and Neck cancer	86	Recruiting	Omega-3 fatty acids
NCT 02330926	No	Gynecological cancers	150	Recruiting	Nutritional supplements Ibuprofen
